# Treatment Selection Based on Dominant Tumor Biology in Endometrial Carcinoma with Choriocarcinomatous Differentiation: A Case-Based Review

**DOI:** 10.3390/curroncol33050251

**Published:** 2026-04-27

**Authors:** Norihito Kamo, Shigenori Furukawa, Asami Kato, Keisuke Yoshida, Chikako Okabe, Hideki Miura, Tetsu Sato, Hiroshi Suzuki, Shu Soeda, Keiya Fujimori

**Affiliations:** 1 Department of Obstetrics and Gynecology, School of Medicine, Fukushima Medical University, Fukushima 960-1295, Japan; 2 Department of Obstetrics and Gynecology, Shirakawa Kosei General Hospital, Fukushima 961-0005, Japan; 3 Department of Obstetrics and Gynecology, Jusendo General Hospital, Fukushima 963-8585, Japan; 4 Department of Regional Gynecologic Oncology, School of Medicine, Fukushima Medical University, Fukushima 960-1295, Japan

**Keywords:** treatment selection, tumor biology, endometrial carcinoma

## Abstract

Endometrial carcinoma (EC) with choriocarcinomatous differentiation is an exceptionally rare malignancy for which standardized postoperative treatment strategies are lacking. Herein, we describe two postmenopausal patients with endometrioid carcinoma containing a choriocarcinomatous component. One patient had an EC-dominant tumor with low and rapidly normalized postoperative serum human chorionic gonadotropin (hCG) levels and received paclitaxel plus carboplatin. The other patient had a choriocarcinomatous-dominant tumor with markedly elevated postoperative serum hCG levels and was treated with gestational trophoblastic neoplasia (GTN)-oriented chemotherapy using etoposide, methotrexate, actinomycin D/cyclophosphamide, and vincristine (EMA/CO). Both patients remain disease-free. A review of representative published cases revealed two competing treatment paradigms, EC-oriented and GTN-oriented, applied inconsistently and without unified selection criteria. On the basis of integrated clinicopathological assessment, we propose that postoperative treatment consideration should be guided primarily by dominant tumor biology, rather than the International Federation of Gynecology and Obstetrics stage alone. Tumors with a dominant choriocarcinomatous component accompanied by elevated postoperative serum hCG levels may benefit from GTN-oriented chemotherapy, whereas EC-dominant tumors with low or normalized hCG levels may benefit from EC-oriented regimens. Reassessment of dominant tumor biology using postoperative pathological findings and serum hCG dynamics may provide a pragmatic, decision-support framework for adjuvant treatment consideration in this rare and clinically challenging entity.

## Simple Summary

Endometrial cancer with choriocarcinomatous differentiation is an extremely rare disease, and gynecologic oncologists often face difficulty deciding the best postoperative treatment. Some tumors behave as an ordinary endometrial cancer tumor, whereas others act more like a choriocarcinoma, a highly aggressive tumor that produces human chorionic gonadotropin. Herein, we describe two patients who had very different tumor behaviors despite having the same diagnosis. One patient was treated successfully with standard chemotherapy for endometrial cancer, whereas the other required chemotherapy typically used for choriocarcinoma. By reviewing these cases and previous reports, we found that treatment decisions may be better guided by the dominant biological behavior of the tumor and postoperative human chorionic gonadotropin levels rather than the disease stage alone. This approach may help clinicians choose more appropriate treatments and improve outcomes in patients with this rare condition.

## 1. Introduction

Endometrial carcinoma (EC) is the most common gynecologic malignancy in developed countries, and standard treatment strategies are established on the basis of the International Federation of Gynecology and Obstetrics (FIGO) stage and histological subtype. However, these algorithms may become inadequate in rare histological variants and mixed tumors, resulting in substantial uncertainty in postoperative treatment selection. EC with choriocarcinomatous differentiation represents one of the most challenging examples of such scenarios, in which conventional stage-based decision-making often fails [1,2,3,4,5,6,7].

EC with choriocarcinomatous differentiation is characterized by the coexistence of epithelial carcinoma and trophoblastic tumor components within a single neoplasm and is exceptionally rare. It has been reported sporadically in the literature, predominantly as single case reports or small case series [2,3,5,6]. In most cases, the choriocarcinomatous component is considered to arise through somatic, non-gestational differentiation of epithelial carcinoma cells, although alternative pathogenetic mechanisms have also been described, contributing to pathological heterogeneity and diagnostic ambiguity [1,4].

Despite these pathological considerations, the most critical unresolved issue is not tumor classification itself but the absence of a clinically applicable framework for postoperative treatment selection. Conventional management of EC relies primarily on FIGO stage-based risk stratification [8,9,10], whereas gestational trophoblastic neoplasia (GTN) is treated according to serum human chorionic gonadotropin (hCG) level-guided, regimen-specific chemotherapy [11,12,13]. Tumors with choriocarcinomatous differentiation lie at the intersection of these two fundamentally different treatment paradigms, rendering the FIGO stage alone insufficient to guide therapeutic decisions [1,11].

A review of previously reported cases indicated that postoperative management has diverged into two main approaches: EC-oriented therapy and GTN-oriented therapy [1,2,5,6]. Importantly, these strategies have been applied inconsistently, without a clear correlation to the FIGO stage, tumor composition, or serum hCG assessment, suggesting that treatment selection has often depended on empirical, case-by-case judgment [1,5]. Furthermore, this disease is rarely diagnosed preoperatively. The presence of a choriocarcinomatous component is difficult to predict on the basis of biopsy or imaging findings alone, and the serum hCG level is seldom assessed before surgery [2,6]. Consequently, preoperative indicators offer limited guidance, underscoring the need for a postoperative, pathology-driven approach to clinical decision making.

Herein, we describe two cases of postmenopausal women with EC with choriocarcinomatous differentiation and re-examine representative previously reported cases. We also propose a pragmatic, biology-driven hypothesis that postoperative treatment consideration should be guided not solely by the FIGO stage but by dominant tumor biology, integrating the pathological tumor composition with postoperative serum hCG dynamics, to clarify clinical reasoning when conventional treatment algorithms are insufficient.

## 2. Detailed Case Description

### 2.1. Case 1

A 52-year-old postmenopausal woman (gravida 2, para 2) presented with abnormal vaginal bleeding. Imaging studies demonstrated a 90-mm intrauterine mass extending toward the cervix, invading less than half of the myometrium, and no apparent lymph node metastasis or distant metastasis. Endometrial biopsy revealed grade 3 endometrioid carcinoma. The preoperative serum hCG level was not assessed. She had no relevant medical or family history and was not taking any medications.

**Figure 1:**
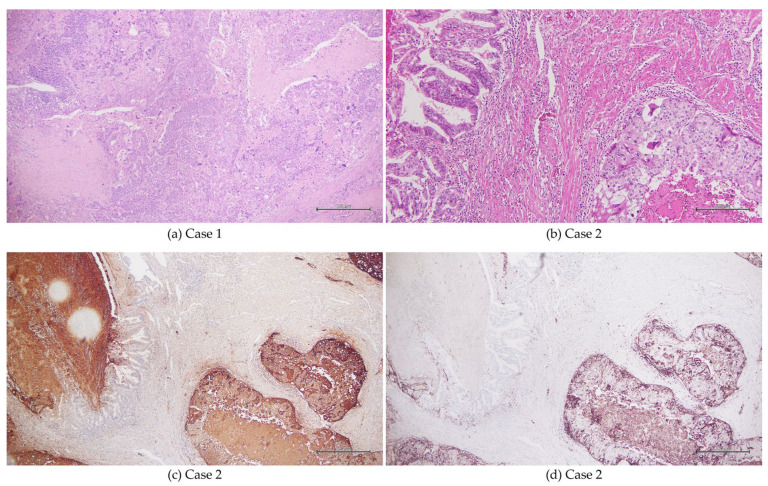
Representative pathological findings in cases 1 and 2. (**a**) Case 1 (hematoxylin and eosin staining, ×40). Approximately 70% of the tumor was composed of high-grade (G3) endometrioid carcinoma exhibiting a solid growth pattern with marked nuclear atypia. The remaining 30% consisted of a choriocarcinomatous component characterized by eosinophilic, multinucleated tumor cells resembling syncytiotrophoblasts. Transitional areas showing admixture of the two components were identified. (**b**) Case 2 (hematoxylin and eosin staining, ×40). Focally, fused glandular structures consistent with a grade 1 endometrioid carcinoma component were observed. In contrast, the predominant tumor component showed solid and infiltrative proliferation of cytotrophoblast-like cells, with scattered multinucleated syncytiotrophoblast-like cells. (**c**) Case 2 (immunohistochemistry for β-human chorionic gonadotropin [β-hCG], ×40). Syncytiotrophoblast-like cells demonstrated positive immunoreactivity for β-hCG. (**d**) Case 2 (immunohistochemistry for programmed death-ligand 1 [PD-L1], ×40). PD-L1 expression was observed in the choriocarcinomatous component.

The patient underwent total abdominal hysterectomy with bilateral salpingo-oophorectomy, along with pelvic and para-aortic lymphadenectomy. Histopathological evaluation revealed a biphasic tumor composed predominantly of grade 3 endometrioid carcinoma, accounting for approximately 70% of the overall tumor volume, with an admixed choriocarcinomatous component. Lymphovascular and venous invasion were present. The final diagnosis was endometrial carcinoma with choriocarcinomatous differentiation, staged as pT2N0M0, corresponding to FIGO 2009 stage II. According to the FIGO 2023 classification, the case is also consistent with stage IIB, based on anatomical findings. However, molecular classification was not available (Figure 1a).

Immunohistochemical analysis demonstrated PD-L1 expression restricted to the choriocarcinomatous component, with weak β-hCG positivity also confined to this component. The postoperative serum hCG level, measured at the time of pathological diagnosis, was 1.9 IU/L (reference range: <5 IU/L) and normalized within 1 month. Given the predominance of the endometrioid carcinoma component, which was also responsible for the invasive growth pattern and lymphovascular invasion, adjuvant chemotherapy with paclitaxel plus carboplatin was administered for six cycles. The patient has remained disease-free for 5 years following treatment completion.

### 2.2. Case 2

A 56-year-old postmenopausal woman (gravida 3, para 2) presented with abnormal uterine bleeding. Endometrial biopsy revealed grade 1 endometrioid carcinoma without evidence of a choriocarcinomatous component. Imaging studies demonstrated a uterine-confined lesion invading less than half of the myometrium and no apparent lymph node or distant metastasis. Similar to the other patient, this patient had no remarkable medical or family history and was not taking any regular medications. She subsequently underwent total abdominal hysterectomy with bilateral salpingo-oophorectomy and pelvic and para-aortic lymphadenectomy.

Histopathological examination revealed a tumor composed predominantly of choriocarcinomatous elements, with a minor component of grade 1 endometrioid carcinoma, and venous invasion was present. The final diagnosis was endometrial carcinoma with choriocarcinomatous differentiation, staged as pT1aN0M0, corresponding to FIGO 2009 stage IA. According to the FIGO 2023 classification, this case is consistent with stage IA, based on anatomical findings. However, molecular classification was not available. No lymph node metastasis was identified on pathological examination. Immunohistochemical analysis demonstrated strong positivity for β-hCG and PD-L1 within the choriocarcinomatous component (Figure 1b–d).

The postoperative serum hCG level measured at the time of pathological diagnosis was markedly elevated at 148.9 IU/L. After confirming the absence of distant metastasis, adjuvant chemotherapy with EMA/CO was initiated. The serum hCG level normalized after three cycles, and a total of six cycles of chemotherapy were completed. The patient remains disease-free 24 months after treatment completion.

Ethical review and approval were waived because it is a case report of two patients encountered in routine clinical practice. Written informed consent has been obtained from the patients to publish this paper.

## 3. Discussion

In the cases described herein, no strict numerical cut-off value was applied to define the dominance of tumor components. Instead, dominant tumor biology was determined through an integrated histopathological assessment incorporating the relative proportion of tumor components across the entire tumor, architectural growth pattern and vascular invasion, and identification of the component primarily responsible for invasive fronts. Each patient’s condition was independently reviewed by at least two board-certified pathologists, and dominance was assigned only when concordant interpretations were achieved through whole-tumor evaluation. This approach is consistent with that employed in prior studies describing the marked pathological heterogeneity of EC with choriocarcinomatous differentiation, including non-endometrioid histologic subtypes [1,4,14].

In cases of EC with choriocarcinomatous differentiation, a major clinical challenge is that the FIGO stage and conventional EC risk stratification alone are often insufficient to guide postoperative treatment selection [1,8,9,10,11]. On the basis of two institutional cases and reinterpretation of previously reported cases, we propose that treatment consideration should be guided by dominant tumor biology, defined as the clinically prevailing tumor behavior assessed through integrated pathological findings and postoperative serum hCG dynamics [1,4]. Strict reliance on FIGO stage alone may obscure clinically relevant biological behavior in selected cases of this rare tumor.

Tumors with a predominant choriocarcinomatous component accompanied by persistently or markedly elevated postoperative serum hCG levels may be more appropriately managed with GTN-oriented chemotherapy than EC-oriented chemotherapy [11,12,13]. Conversely, when the endometrioid carcinoma component is dominant and postoperative serum hCG levels are low or rapidly normalize, conventional EC-oriented chemotherapy seems reasonable [2,3,5,6,7]. Importantly, this framework does not propose a new treatment rule but rather makes explicit how experienced clinicians already deviate from FIGO stage-based algorithms when postoperative tumor biology clearly contradicts stage-based risk assumptions. Although both EC-oriented chemotherapy (e.g., paclitaxel plus carboplatin) and GTN-oriented chemotherapy (e.g., EMA/CO) are effective in selected cases, their toxicity profiles differ substantially. EMA/CO is generally associated with higher hematologic toxicity, hepatotoxicity, and mucosal adverse events, whereas paclitaxel plus carboplatin is more commonly associated with peripheral neuropathy and moderate myelosuppression. Therefore, inappropriate selection of GTN-oriented chemotherapy in tumors that do not exhibit choriocarcinomatous-dominant biology may result in unnecessary treatment-related toxicity. This further supports the importance of tailoring postoperative treatment strategies based on dominant tumor biology rather than applying uniform treatment approaches.

**Figure 2:**
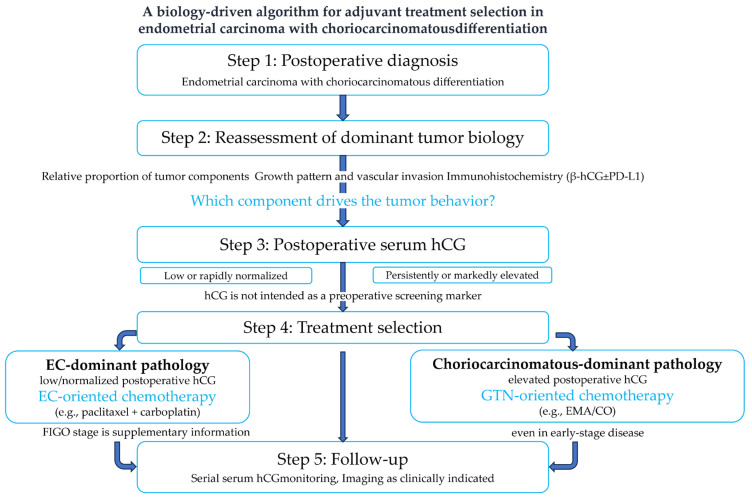
Conceptual, biology-driven framework illustrating how postoperative tumor biology may override the International Federation of Gynecology and Obstetrics (FIGO) stage-based assumptions in the treatment consideration for endometrial carcinoma with choriocarcinomatous differentiation.

Figure 2 illustrates a conceptual decision-support framework rather than a prescriptive treatment algorithm for postoperative treatment consideration in patients with EC with choriocarcinomatous differentiation. The framework emphasizes reassessment of dominant tumor biology following postoperative diagnosis, rather than reliance on FIGO stage alone.

Dominant tumor biology was evaluated through an integrated assessment of the relative proportion of tumor components across the entire tumor, architectural growth pattern and vascular invasion, immunohistochemical findings (β-hCG with or without PD-L1), and postoperative serum hCG dynamics. This assessment was intended to be performed in a multidisciplinary context, incorporating pathological and clinical judgments. In patients with EC-dominant pathology and low or rapidly normalized postoperative serum hCG levels, EC-oriented chemotherapy (e.g., paclitaxel plus carboplatin) may be considered appropriate. Conversely, in patients with choriocarcinomatous-dominant pathology accompanied by persistently or markedly elevated postoperative serum hCG levels, GTN-oriented chemotherapy (e.g., EMA/CO) may be considered. Postoperative serum hCG is not intended as a preoperative screening marker, nor as a single-point determinant of treatment strategy, but rather as an adjunctive biomarker interpreted in conjunction with pathological findings and its postoperative trend, considering the biological half-life of hCG to avoid misinterpretation of transient postoperative elevation. The FIGO stage is regarded as supplementary information and does not solely determine treatment selection, even in early-stage disease.

Our two cases illustrate this concept. In case 1, approximately 70% of the tumor consisted of grade 3 endometrioid carcinoma, which also accounted for the invasive growth pattern and lymphovascular invasion. Postoperative serum hCG levels were low and rapidly normalized, and EC-oriented chemotherapy resulted in long-term disease-free survival despite FIGO stage II classification. However, case 2 comprised predominantly choriocarcinomatous elements and was associated with markedly elevated postoperative serum hCG levels. Although classified as FIGO stage IA, the tumor exhibited GTN-like biological behavior, and GTN-oriented chemotherapy led to rapid hCG normalization and a favorable outcome.

### 3.1. Interpretation of Postoperative Serum hCG Levels

In our cases, the postoperative serum hCG level was measured at the time of the final pathological diagnosis and interpreted on the basis of its temporal trend rather than a single absolute value. Given the short biological half-life of hCG, transient postoperative elevation was distinguished from tumor-driven production by confirming either rapid normalization or persistent elevation on serial measurements.

Reinterpretation of previously reported cases (Table 1) suggests that neither tumor composition nor serum hCG levels alone fully define tumor behavior. Favorable outcomes with GTN-oriented therapy are not uniform, particularly in advanced-stage disease [1,5,6], whereas EC-oriented regimens have achieved long-term survival in EC-dominant tumors even at advanced stages [2,3,7]. As dominance assessment is inherently continuous and partially subjective, treatment consideration should integrate histopathological findings, vascular invasion, imaging findings, and overall clinical context.

Within our institution, GTN-oriented chemotherapy is prioritized when postoperative evaluation demonstrates choriocarcinomatous-dominant pathology with clearly elevated serum hCG levels, whereas EC-oriented regimens are selected when endometrioid carcinoma is dominant and postoperative hCG levels are low or rapidly normalize. Serum hCG should be regarded as an adjunctive biomarker rather than a definitive determinant, as its levels may be influenced by the timing of the measurement and intratumoral heterogeneity [11,15]. A recent systematic review by Mangla et al. demonstrated that trophoblastic differentiation and β-hCG secretion in somatic malignancies of the uterine corpus are associated with adverse prognosis, supporting the biological relevance of the hCG level as a marker of aggressive tumor behavior rather than a purely diagnostic indicator [15]. Their findings reinforce our approach of incorporating postoperative serum hCG dynamics into treatment consideration while avoiding reliance on a single absolute value.

Marked morphological heterogeneity represents a fundamental challenge in tumors with choriocarcinomatous differentiation. Xiao et al. highlighted the wide spectrum of endometrial morphological changes observed in choriocarcinoma specimens, underscoring the diagnostic complexity and potential for sampling bias [16]. These observations are consistent with our emphasis on whole-tumor evaluation and integrated histopathological assessment rather than on strict numerical thresholds when determining dominant tumor biology.

Although PD-L1 expression was observed in the choriocarcinomatous component in our patients, it was not incorporated into treatment decision making. Although emerging data suggest potential roles for immune checkpoint inhibitors in trophoblastic disease [17,18,19,20,21,22], their clinical relevance in EC with choriocarcinomatous differentiation remains unclear. At present, PD-L1 expression alone does not provide sufficient evidence to guide postoperative treatment selection in this rare and heterogeneous disease entity.

### 3.2. Limitations

Some limitations should be considered when interpreting the findings. Comprehensive molecular pathological analyses, including The Cancer Genome Atlas molecular classification, mismatch repair status, p53 abnormalities, and POLE mutations, were not performed [23]. Although molecular classification plays an increasingly important role in the contemporary management of EC, tumors with choriocarcinomatous differentiation are characterized by marked intratumoral heterogeneity. In such biphasic tumors, molecular information derived from a single sampled area may not accurately reflect the overall tumor behavior. Accordingly, we intentionally did not position molecular stratification as the central determinant of treatment consideration, instead proposing a pragmatic framework integrating postoperative pathological tumor composition with serum hCG dynamics. Molecular analyses remain an important area for future investigation and may complement the framework proposed here.

We reported just two cases. The objective was not to generalize treatment outcomes or establish the superiority of any specific chemotherapy regimen but to illustrate the clinical reasoning process underlying treatment consideration in a disease entity where standardized guidance is lacking. These cases were selected to contrast the FIGO stage and dominant tumor biology; therefore, our findings should be regarded as hypothesis-generating rather than definitive.

Finally, reinterpretation of previously reported cases is limited by the heterogeneity and incomplete reporting inherent in the existing literature, particularly with respect to serum hCG measurement and treatment selection criteria. Nevertheless, given the extreme rarity of this disease and the absence of prospective datasets, we sought to extract clinically relevant insights by re-examining published cases through the lens of dominant tumor biology.

Taken together, these limitations indicate that the proposed framework should be viewed as a preliminary, decision-support concept requiring further refinement and validation. However, in a clinical context where treatment decisions have historically relied on empirical judgment, this study provides a structured starting point for postoperative treatment consideration in this rare and complex entity. Both patients were followed regularly after treatment, with no evidence of disease recurrence at the last follow-up [5 years (case 1) and 24 months (case 2)].

## 4. Conclusions

EC with choriocarcinomatous differentiation is a rare tumor that does not fit conventional EC- or GTN-based treatment paradigms. Our findings suggest that postoperative treatment should be guided by dominant tumor biology, integrating pathological tumor composition and postoperative serum hCG dynamics, rather than relying solely on FIGO stage. Choriocarcinomatous-dominant tumors with elevated postoperative hCG levels may benefit from GTN-oriented chemotherapy, whereas EC-dominant tumors with low or normalized hCG levels may be managed with EC-oriented regimens. This biology-driven approach may help optimize treatment selection in this rare and clinically challenging entity.

## Figures and Tables

**Table 1:** Reported cases of endometrial carcinoma with choriocarcinomatous differentiation, highlighting the heterogeneity in dominant tumor components, serum hCG assessment, treatment paradigms, and clinical outcomes.

Author (y)	No. of Cases	Age (y)/ Menopause Status	FIGO Stage	Dominant Tumor Component	Serum hCG (Timing)	Treatment Paradigm	Chemotherapy Regimen	Role of Surgery	PD-L1 Expression	Outcome
Yamada et al. (2009) [2]	1	52/Post	IIIC	Endometrioid > CC	Not reported	EC-oriented	TC	Primary surgery	Not reported	DOD
Ishida & Okabe (2013) [3]	1	55/Post	IB	Endometrioid > CC	Elevated (post-op)	EC-oriented	TC	Primary surgery	Not reported	NED
Wakahashi et al. (2012) [7]	1	63/Post	II	Mixed	Not reported	EC-oriented	TC	Primary surgery	Not reported	AWD
Xie et al. (2022) [6]	1	31/Pre	IIIC	CC > Endometrioid	Elevated (pre-op)	GTN-oriented	EMA/CO	Primary surgery	Not reported	NED
Bai et al. (2023) [5]	1	59/Post	IVB	CC > Endometrioid	Markedly elevated	GTN-oriented	EMA/CO	Palliative	Not reported	DOD
Present cases	2	Post	IA, II	EC-dominant/ CC-dominant	1.9; 148.9 IU/mL (post-op)	EC-oriented/GTN-oriented	TC/EMA-CO	Primary surgery	Positive (restricted to choriocarcinomatous component)	NED (5 y/24 mo)

EC-oriented indicates management primarily based on conventional endometrial carcinoma protocols (e.g., paclitaxel plus carboplatin). GTN-oriented indicates management based on gestational trophoblastic neoplasia regimens (e.g., EMA/CO). Serum hCG levels were reported heterogeneously across studies with respect to timing and clinical context, limiting direct comparison. In the present cases, serum hCG levels were measured postoperatively at the time of pathological diagnosis and interpreted on the basis of their postoperative trend. PD-L1 expression was assessed using immunohistochemistry when reported. CC, choriocarcinomatous component; NED, no evidence of disease; AWD, alive with disease; DOD, died of disease; Post, postmenopausal; Pre, premenopausal; post-op, postoperatively; pre-op, preoperatively; PD-L1, programmed death-ligand 1; EC, endometrial carcinoma; GTN, gestational trophoblastic neoplasia; no., number. TC, paclitaxel plus carboplatin; EMA/CO, etoposide, methotrexate, actinomycin D/cyclophosphamide, vincristine.

## Data Availability

The data presented in this study are available upon request from the corresponding author due to concerns regarding patient privacy.

## Abbreviations

The following abbreviations are used in this manuscript:

FIGO	International Federation of Gynecology and Obstetrics
hCG	Human chorionic gonadotropin
EC	Endometrial carcinoma
GTN-oriented	Gestational trophoblastic neoplasia-oriented
β-hCG	β-human chorionic gonadotropin
PD-L1	Programmed death-ligand 1
